# Retrospective Analysis of In-Stent Restenosis in Diabetic Patients

**DOI:** 10.7759/cureus.90441

**Published:** 2025-08-18

**Authors:** Haseeba Khalid, Hammad Mudassar, Ali Husnain, Muhammad Fakhar Hayat, Babar Jahangir Baig, Atif Nazir, Hamza Riaz

**Affiliations:** 1 Internal Medicine, Akhtar Saeed Medical and Dental College, Lahore, PAK; 2 Cardiology, Shalamar Hospital, Lahore, PAK; 3 Internal Medicine, Sheikh Zayed Hospital, Lahore, PAK; 4 Cardiology, Rawalpindi Institute of Cardiology, Rawalpindi, PAK; 5 Internal Medicine, Nawaz Sharif Social Security Hospital, Lahore, PAK

**Keywords:** angiography, diabetics, drug-eluting stents, in-stent restenosis, percutaneous coronary intervention

## Abstract

Background

In-stent restenosis (ISR) remains a major clinical challenge in diabetic patients undergoing percutaneous coronary intervention (PCI), despite the widespread use of drug-eluting stents (DES).

Objective

This study aims to determine the incidence of ISR and identify associated clinical and procedural risk factors in diabetic patients following PCI with DES.

Materials and methods

This retrospective study was conducted at Akhtar Saeed Trust Hospital, Lahore, Pakistan, from April 2023 to April 2025. A total of 265 diabetic patients who underwent PCI with DES implantation and later presented for follow-up angiographic evaluation were enrolled in the study. Participants were selected through non-probability consecutive sampling. Data were collected using a structured proforma, which included demographic information (age, sex), clinical history (duration of diabetes, smoking status, hypertension, dyslipidemia), laboratory parameters (HbA1c, fasting glucose, lipid profile), and procedural details (number, type, and length of stents used; lesion characteristics; and target vessel).

Results

ISR was observed in 76 out of 265 patients (28.7%). Patients with ISR had significantly higher HbA1c levels (8.6 ± 1.2% vs. 7.3 ± 1.1%, p < 0.001), longer duration of diabetes (11.1 ± 4.3 vs. 8.4 ± 4.2 years, p < 0.001), and more frequent use of long stents and smaller stent diameters. Multivariate regression identified poor glycemic control (OR 2.83, p < 0.001), diabetes duration > 10 years (OR 2.45, p = 0.005), and stent length >28 mm (OR 1.96, p = 0.015) as independent predictors of ISR. ISR patients were also more symptomatic at follow-up.

Conclusions

ISR remains a frequent and serious complication in diabetic patients after PCI. Poor glycemic control, longer disease duration, and certain stent characteristics significantly contribute to its development.

## Introduction

In-stent restenosis (ISR) is defined as the re-narrowing of a coronary artery at the site of a previously placed stent, typically due to neointimal hyperplasia or neoatherosclerosis, resulting in a ≥50% reduction in luminal diameter [1]. Although technological breakthroughs, especially the use of drug-eluting stents (DES), have decreased the incidence of ISR by a significant margin, it remains a significant clinical problem, especially in patients with diabetes mellitus (DM). Even in the second-generation DES, diabetic patients have almost twice the frequency of ISR as non-diabetics [2].

This augmented predisposition is not by chance but as a result of a set of metabolic, vascular, and cellular abnormalities, which are characteristic features of diabetes [3]. Some of them are continuous hyperglycemia, insulin resistance, augmented oxidative stress, systemic inflammation, and degraded endothelial repair, all of which lead to hyperexaggerated neointimal hyperplasia and hindrance to vascular treatment occurring after PCI.

The defect, aberrant growth, and translocation of vascular smooth muscle cells (VSMCs) caused by high concentrations of advanced glycation end products (AGEs) and pro-inflammatory cytokines lie among the most powerful mechanisms of ISR in diabetic patients [4]. Hyperglycemia enhances the protein kinase C pathway and induces a growth factor, which includes vascular endothelial growth factor, platelet-derived growth factor, and TGF-β, which in turn contribute to the damage of the intima layer. In addition, glycation of the extracellular matrix structures causes stiff vessels, which tend towards undesirable remodeling at the stent point [5]. Added to this is the issue of microvascular disease and diffuse disease of the coronary arteries in the diabetic patient, which is more resistant to complete revascularization and more prone to restenosis despite the technically best stenting effort [6].

ISR in diabetic patients is a clinically significant complication despite the utilization of the newer generation DES, presenting high-quality anti-proliferative coating and enhanced polymer biocompatibility [7]. It has already been established in several studies that diabetic patients, and more specifically patients with insulin-dependent DM, have a worse outcome after stenting with a greater frequency of target lesion revascularization, myocardial infarction, and even mortality [8]. Furthermore, ISR susceptibility seems to depend on levels of glycemic control. Ineffective management of HbA1c is always linked with high ISR rates, and this is why it is worth paying much attention to intensive glycemic control both pre- and post-PCI [9]. ISR may have a wide range of clinical manifestations, ranging from a silent ischemia on routine follow-up stress testing to an acute coronary syndrome [10]. ISR in diabetic patients is multifaceted to manage. It consists of a change of lifestyle and risk factors, optimization of antiplatelet therapy, intervention revascularization with repeat DES implantation or drug-coated balloon angioplasty, and, in some cases, coronary artery bypass grafting (CABG) of diffuse disease [11].

It is essential to individualize therapy depending on the etiology of ISR based on lesion characteristics, the type of stent being used, and the comorbidities of a patient [12]. Adjunctive treatments, such as the use of anti-proliferative agents, new stent platforms, and improved metabolic control, are being reviewed to reduce the incidence of ISR in diabetics and prevent it altogether [13].

This study aims to determine the incidence of ISR and identify associated clinical and procedural risk factors in diabetic patients following PCI with DES.

## Materials and methods

Methodology

This retrospective study was conducted at Akhtar Saeed Trust Hospital, Lahore, Pakistan, from April 2023 to April 2025. The Institutional Review Board of Akhtar Saeed Medical and Dental College issued approval 04-AMDC/ADM/2022. A total of 265 diabetic patients who underwent PCI with DES implantation and later presented for follow-up angiographic evaluation were enrolled in the study. Participants were selected through non-probability consecutive sampling. All eligible diabetic patients undergoing follow-up coronary angiography, either symptom-driven or routine, were considered for inclusion.

Inclusion and exclusion criteria

Patients included in the study were those diagnosed with type 2 DM according to the American Diabetes Association criteria, with a history of PCI using DES performed at least six months prior to the study. Eligible participants were required to have complete clinical, biochemical, and angiographic records and be between 30 and 75 years of age. Patients were excluded if they had type 1 DM, a history of CABG, evidence of stent thrombosis, stent fracture, or malapposition on imaging, or chronic total occlusion at the stent site.

Data collection

Data were collected using a structured proforma, which included demographic information (age, sex), clinical history (duration of diabetes, smoking status, hypertension, dyslipidemia), laboratory parameters (HbA1c, fasting glucose, lipid profile), and procedural details (number, type, and length of stents used; lesion characteristics; and target vessel). Follow-up coronary angiography was performed to assess the presence and severity of ISR.

Definition of ISR

ISR was defined as luminal narrowing of ≥ 50% at the stented segment or its 5-mm edges, detected by quantitative coronary angiography.

Statistical analysis

Data were analyzed using SPSS Statistics version 26 (IBM Corp. Released 2019. IBM SPSS Statistics for Windows, Version 26.0. Armonk, NY: IBM Corp.). Independent t-tests were used to compare continuous variables, which were expressed as mean standard deviation (SD). Categorical variables were expressed as frequencies and percentages and compared using the chi-square test. Independent predictors of ISR were found using multivariate logistic regression. A p-value of <0.05 was considered statistically significant.

## Results

Data were collected from 265 patients. The baseline characteristics of the ISR and non-ISR groups showed no significant differences in age or gender distribution, with mean ages of 60.1 ± 8.2 years for the ISR group and 59.0 ± 9.1 years for the non-ISR group, and 67.1% males in the ISR group and 66.1% males in the non-ISR group. However, the ISR group had a significantly longer duration of diabetes (11.1 ± 4.3 years) compared to the non-ISR group (8.4 ± 4.2 years), with a p-value < 0.001. Additionally, the ISR group had a higher mean HbA1c (8.6 ± 1.2%) compared to the non-ISR group (7.3 ± 1.1%), suggesting poorer glycemic control in the ISR group. Hypertension and dyslipidemia were prevalent in both groups, with the ISR group having slightly higher rates of these comorbidities (71.0% and 76.3%, respectively) compared to the non-ISR group (68.8% and 66.1%, respectively) (Table 1).

**Table 1:** Baseline demographic and clinical characteristics of study participants (n = 265) Data are represented as mean ± SD or percentages (%). DM: diabetes mellitus, ISR: in-stent restenosis, SD: standard deviation

Variable	ISR group (n = 76)	Non-ISR group (n = 189)
Age (years)	60.1 ± 8.2	59.0 ± 9.1
Male gender, n (%)	51 (67.1%)	125 (66.1%)
Duration of DM (years)	11.1 ± 4.3	8.4 ± 4.2
HbA1c (%)	8.6 ± 1.2	7.3 ± 1.1
Hypertension, n (%)	54 (71.0%)	130 (68.8%)
Dyslipidemia, n (%)	58 (76.3%)	125 (66.1%)

The ISR group had a higher prevalence of multivessel disease (59.2%) compared to the non-ISR group (41.1%) (p = 0.008). The ISR group also had a significantly higher proportion of long stents (length > 28 mm), with 55.3% compared to 33.0% in the non-ISR group (p = 0.002), and smaller stent diameters (< 2.75 mm), with 52.6% in the ISR group compared to 33.9% in the non-ISR group (p = 0.01). However, the placement of stents in the left anterior descending (LAD) artery did not differ significantly between the two groups (64.5% vs. 55.5%, p = 0.18) (Table 2).

**Table 2:** Angiographic and procedural characteristics Data are represented as percentages (%). Significance level set at p < 0.05. LAD: left anterior descending, ISR: in-stent restenosis

Variable	ISR group (n = 76)	Non-ISR group (n = 189)	p-value
Multivessel disease, n (%)	45 (59.2%)	78 (41.1%)	0.008
Long stent (> 28 mm), n (%)	42 (55.3%)	62 (33.0%)	0.002
Small stent diameter (< 2.75 mm), n (%)	40 (52.6%)	64 (33.9%)	0.01
Stent in LAD, n (%)	49 (64.5%)	105 (55.5%)	0.18

**Figure 1:**
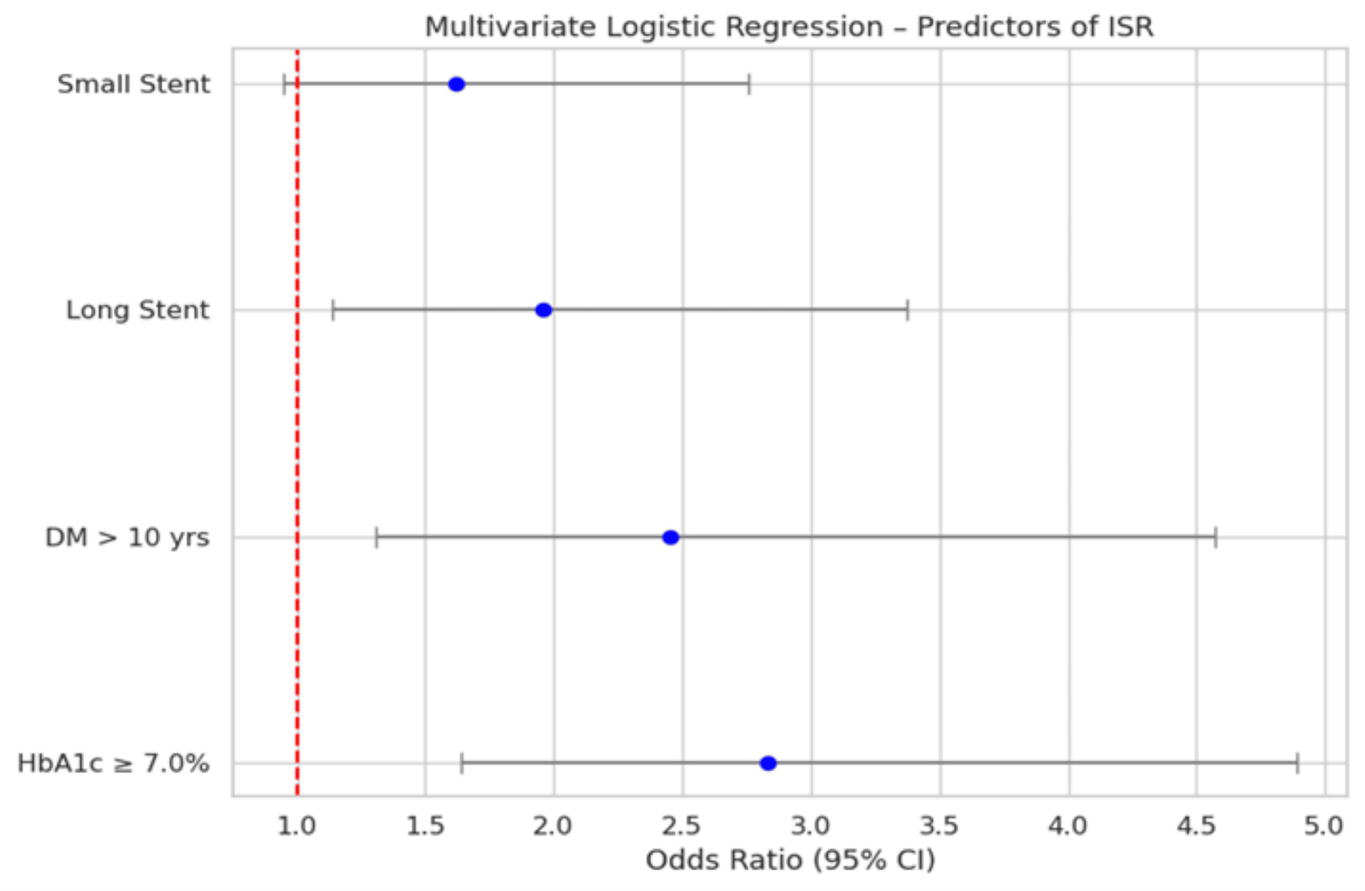
Multivariate logistic regression analysis for predictors of ISR ISR: in-stent restenosis, DM: diabetes mellitus, HbA1c: glycated hemoglobin, CI: confidence interval

Higher HbA1c levels (≥ 7.0%) were associated with 2.83 times higher odds of ISR (p < 0.001). A longer duration of diabetes (more than 10 years) also increased the odds of ISR, with an odds ratio (OR) of 2.45 (p = 0.005). The use of long stent lengths (> 28 mm) was another significant predictor, with an OR of 1.96 (p = 0.015). While smaller stent diameters (< 2.75 mm) showed an increased likelihood of ISR (OR = 1.62), this association did not reach statistical significance (p = 0.07) (Table 3, Figure 1).

**Table 3:** Multivariate logistic regression analysis for predictors of ISR ORs with 95% CI are reported. Significance level set at p < 0.05. DM: diabetes mellitus, OR: odds ratio, CI: confidence interval

Predictor	OR	95% CI	p-value
HbA1c ≥ 7.0%	2.83	1.64-4.89	<0.001
Duration of DM > 10 years	2.45	1.31-4.57	0.005
Long stent length (> 28 mm)	1.96	1.14-3.37	0.015
Small stent diameter (< 2.75 mm)	1.62	0.95-2.76	0.07

Medication usage at follow-up showed that a high proportion of both groups were on dual antiplatelet therapy (DAPT), statins, and ACE inhibitors/ARBs, with no significant differences between the ISR and non-ISR groups (p = 0.31, 0.21, and 0.88, respectively). However, the ISR group had a significantly higher proportion of patients on insulin therapy (64.5% vs. 46.6%, p = 0.01), indicating more advanced or poorly controlled diabetes in this subgroup. The use of beta-blockers was similar between the groups (71.1% in the ISR group vs. 73.0% in the non-ISR group, p = 0.76) (Table 4).

**Table 4:** Medication usage at follow-up A significantly higher proportion of ISR patients were on insulin therapy (p = 0.01), suggesting more advanced or poorly controlled diabetes in this subgroup. ACE: angiotensin converting enzyme, ARB: angiotensin-II receptor blocker, ISR: in-stent restenosis, DAPT: dual antiplatelet therapy

Medication	ISR group (n = 76)	Non-ISR group (n = 189)	p-value
DAPT, n (%)	72 (94.7%)	184 (97.4%)	0.31
Statins, n (%)	65 (85.5%)	172 (91.0%)	0.21
Beta-blockers, n (%)	54 (71.1%)	138 (73.0%)	0.76
ACE inhibitors/ARBs, n (%)	58 (76.3%)	146 (77.2%)	0.88
Insulin therapy, n (%)	49 (64.5%)	88 (46.6%)	0.01

**Figure 2:**
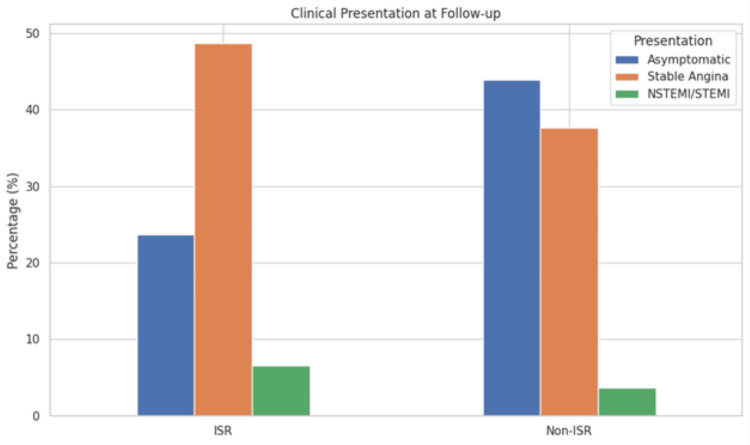
Distribution of clinical presentations between ISR and non-ISR groups ISR: in-stent restenosis, STEMI: ST-segment elevation myocardial infarction, NSTEMI: non-ST-segment elevation myocardial infarction

At follow-up, a significantly higher proportion of patients in the ISR group were asymptomatic (23.7%) compared to the non-ISR group (43.9%) (p = 0.003). The proportion of patients with stable angina was higher in the ISR group (48.7%) compared to the non-ISR group (37.6%), though this difference was not statistically significant (p = 0.09). The rates of unstable angina, NSTEMI/STEMI, and other clinical presentations were similar between the two groups, with p-values of 0.20 and 0.33, indicating no significant differences in the severity of clinical outcomes between the groups (Table 5, Figure 2).

**Table 5:** Clinical presentation at follow-up ISR patients were significantly less likely to be asymptomatic and more likely to present with symptoms or ischemia (p = 0.003). ISR: in-stent restenosis, STEMI: ST-segment elevation myocardial infarction, NSTEMI: non-ST-segment elevation myocardial infarction

Presentation type	ISR group (n = 76)	Non-ISR group (n = 189)	p-value
Asymptomatic, n (%)	18 (23.7%)	83 (43.9%)	0.003
Stable angina, n (%)	37 (48.7%)	71 (37.6%)	0.09
Unstable angina, n (%)	16 (21.1%)	28 (14.8%)	0.20
NSTEMI/STEMI, n (%)	5 (6.6%)	7 (3.7%)	0.33

## Discussion

This study investigated the incidence and risk factors of ISR among diabetic patients following PCI with DES. The observed ISR rate of 28.7% is significantly higher than reported in non-diabetic populations, aligning with previous studies that highlight the adverse vascular remodeling and impaired healing responses characteristic of DM. In a study by Tada et al., insulin-treated diabetics exhibited a significantly higher ISR rate compared to both non-insulin-treated diabetics and non-diabetics, reinforcing the role of metabolic status in restenosis risk [3]. Similarly, Zhao et al. reported increased long-term ISR rates in diabetics following repeat DES implantation, indicating the persistent challenge of maintaining long-term stent patency in this subgroup [5].

One of the most notable findings in our study was the strong association between poor glycemic control and ISR. Patients with elevated HbA1c levels had nearly three times the odds of developing ISR, consistent with studies by Yang et al. and Yi et al., who demonstrated that both absolute HbA1c levels and glycemic variability were independent predictors of restenosis [13,14]. Chronic hyperglycemia promotes neointimal proliferation, oxidative stress, and impaired endothelial recovery mechanisms central to the pathogenesis of ISR in diabetes. This highlights the critical importance of optimal glycemic control both before and following PCI to reduce the risk of restenosis [15]. Stent-related factors also contributed to ISR development. Long stent length (> 28 mm) and smaller vessel diameter (< 2.75 mm) were more prevalent among ISR patients. This is consistent with existing literature, which indicates that long lesion coverage and smaller vessels, typically observed in diabetics due to diffuse and distal coronary artery disease, are strong predictors of restenosis [16]. These anatomical challenges necessitate careful pre-procedural planning, with attention to stent sizing, lesion preparation, and post-dilation techniques to minimize the mechanical triggers of restenosis. Longer stents inherently cover more vessel surface, increasing the risk of delayed endothelialization and neointimal hyperplasia, while smaller vessels provide less luminal reserve, amplifying the impact of any tissue proliferation.

Insulin therapy was more commonly observed in the ISR group, suggesting a link between advanced diabetes and poor vascular outcomes [17]. Even though the literature has not yet come to a consensus on whether or not insulin per se is involved in restenosis, the consensus is that insulin therapy is the expression of more advanced or worse-controlled diabetes. It is conceivable that the elevated rates of ISR in insulin-treated patients further indicate the burden of systemic vascular disease and metabolic perturbation [18]. Clinically, ISR patients were more likely to be symptomatic at follow-up, particularly with stable angina. This highlights the importance of constant follow-up and non-invasive surveillance methods in diabetic patients after PCI and in patients with multiple risk factors. Our results are in line with observations by Schwalm et al., who reported that many ISR cases present later with angina or myocardial infarction despite adherence to antiplatelet therapy [8]. Angiographic evaluation, however, may not be as accurate as using additional tools, including intravascular ultrasound or optical coherence tomography, to estimate the correct diagnosis of the stent patency and additional management [19].

Despite the established effectiveness of the second-generation DES in the whole population, they are less effective in diabetic individuals. This needs a multifactorial management approach, which goes past revascularization only [20,21]. Pharmacologic and lifestyle interventions, along with strict metabolic control, are important elements. Further, there are promising new treatments aimed at vascular inflammation, endothelial repair, and glycemic variability as a means of alleviating the risk of ISR among diabetics. To conclude, this paper demonstrates that ISR remains a clinical challenge in diabetic patients even during the interventional age of modern medicine. The results indicate that more personal and aggressive treatment approaches should be taken to achieve better long-term effects in a high-risk group. Future research should explore targeted interventions that address the unique vascular biology of diabetes, as well as the role of novel devices and systemic therapies in preventing restenosis.

This study has several limitations. Being a single-center retrospective analysis, there is potential for selection and information bias. The lack of intravascular imaging (such as optical coherence tomography or intravascular ultrasound) limits precise assessment of stent apposition and neointimal characteristics. We did not include a non-diabetic comparison group, which would have provided additional insight into the relative risk posed by diabetes. Medication adherence post-discharge was not objectively verified, and the follow-up period may not fully capture late ISR cases. Lastly, angiographic follow-up was performed based on symptoms or clinical indication rather than routinely, which may underestimate the true ISR prevalence.

## Conclusions

ISR remains a prevalent and clinically significant complication among diabetic patients undergoing PCI, despite the use of advanced DES. This study demonstrated a high ISR rate of 28.7%, with poor glycemic control, longer duration of diabetes, long stented segments, and small vessel diameter emerging as key contributing factors. Insulin therapy was also associated with increased ISR risk, likely reflecting more advanced disease. These findings reinforce the complex interplay between metabolic dysregulation and vascular healing in diabetes, underscoring the limitations of current stent-based solutions in this high-risk group.
